# Hypophosphatemia in Dogs With Presumptive Sepsis: A Retrospective Study (2008–2018)

**DOI:** 10.3389/fvets.2021.636732

**Published:** 2021-03-08

**Authors:** Victoria Chu, Robert Goggs, Allison Bichoupan, Shalini Radhakrishnan, Julie Menard

**Affiliations:** Department of Clinical Sciences, College of Veterinary Medicine, Cornell University, Ithaca, NY, United States

**Keywords:** phosphate, sepsis, canine, critical illness, veterinary

## Abstract

**Background:** In humans with sepsis, hypophosphatemia is a marker of illness severity and a negative prognostic indicator. Hypophosphatemia has not been previously investigated in dogs with sepsis, however. This study aimed to estimate the prevalence of hypophosphatemia in dogs, the prevalence of presumptive sepsis in dogs with hypophosphatemia, the prevalence of hypophosphatemia in dogs with presumptive sepsis and the association between outcome and hypophosphatemia in dogs with presumptive sepsis.

**Methods:** Electronic medical records of the Cornell University Hospital for Animals from 2008–2018 were queried to identify all dogs with hypophosphatemia and all dogs with presumptive sepsis. Hypophosphatemia was defined as a serum phosphate concentration <2.7 mg/dL. Sepsis was presumed where ≥2 of 4 systemic inflammatory response syndrome (SIRS) criteria were satisfied associated with a documented or highly suspected infection. Variables were assessed for normality using the D'Agostino-Pearson test. Continuous variables were compared between groups using the Mann-Whitney *U* test. Differences in frequency between categorical variables were analyzed using contingency tables, calculation of Fisher's exact test or Chi^2^ and estimation of odds ratios.

**Results:** In the study period, 47,992 phosphate concentration measurements from 23,752 unique dogs were identified. After eliminating repeat analyses, the period prevalence of hypophosphatemia on a per dog basis over the 11-year study period was 10.6% (2,515/23,752). The prevalence of presumptive sepsis within dogs with hypophosphatemia was 10.7% (268/2,515). During the 11-year study period, 4,406 dogs with an infection were identified, of which 1,233 were diagnosed with presumptive sepsis and had a contemporaneous phosphate concentration. Hypophosphatemia was more prevalent in dogs with presumptive sepsis than in dogs without 21.7 vs. 10.2%; OR 2.44 [95% CI 2.12–2.81]; *P* < 0.0001. The mortality rate was greater in dogs with hypophosphatemia and presumptive sepsis than in dogs with hypophosphatemia without presumptive sepsis (15.3 vs. 3.1%; OR 5.70 [95% CI 3.76–8.52]; *P* < 0.0001), however hypophosphatemia was not associated with outcome in dogs with presumptive sepsis OR 0.87 [95% CI 0.60–1.26]; *P* = 0.518.

**Conclusions:** In dogs with hypophosphatemia, a presumed diagnosis of sepsis was associated with increased mortality compared to other associated disease processes. In dogs with presumptive sepsis, hypophosphatemia was not associated with outcome.

## Introduction

Phosphorus is fundamental for bone mineralization, membrane integrity, metabolism, and cellular signaling (1, 2). Approximately 85% of total body phosphorus exists as hydroxyapatite in bone and teeth; while 14% is intracellular within phospholipids, phosphoproteins, nucleic acids, enzymes, cofactors, and biochemical intermediates. Less than 1% of total body phosphate occurs as free phosphate ions in plasma, known as inorganic phosphate (3). Body phosphorus balance occurs through cholecalciferol and parathyroid hormone-mediated intestinal absorption, renal excretion, and mobilization from bone also controlled by parathyroid hormone (4).

Hypophosphatemia in humans is broadly defined as serum phosphorus (inorganic phosphate) concentrations <2.5 mg/dL with severe hypophosphatemia considered <1.0 mg/dL (5). Clinical signs of mild hypophosphatemia include generalized weakness, anorexia, and disorientation, while severe hypophosphatemia can induce life-threatening cardiac arrhythmias, acute respiratory failure, hemolysis, seizures, or coma (3, 6). In humans, hypophosphatemia has been associated with diabetic ketoacidosis (DKA), head trauma, refeeding syndrome, hypothermia, acute liver failure and sepsis (7). In dogs, hypophosphatemia has been associated with DKA, refeeding syndrome, and hyperparathyroidism (8–11).

The causes of hypophosphatemia in critical illness are multifactorial (4). Hypophosphatemia occurs in 60–80% of critically ill humans with sepsis (12), particularly that due to Gram-negative bacteria (2). In humans with sepsis, hypophosphatemia is a marker of illness severity (13) and a negative prognostic indicator (3). Canine sepsis carries a guarded prognosis with reported mortality rates of 20–68% (14–16). Numerous prognostic indicators have been identified, such as degenerative left shift, inadequate lactate clearance, and various laboratory biomarkers (15, 17–22), but the association between phosphate concentration and outcome has not been investigated. Likewise, the prevalence of hypophosphatemia in dogs with sepsis is unknown. Thus, this study aimed to evaluate the overall incidence of hypophosphatemia in dogs undergoing serum biochemistry testing, determine the prevalence of hypophosphatemia in dogs with presumptive sepsis, and evaluate if hypophosphatemia is associated with outcome. It was hypothesized that hypophosphatemia is more prevalent in dogs with presumptive sepsis than in dogs with other disease processes and that hypophosphatemia is associated with increased mortality in dogs with presumptive sepsis.

## Materials and Methods

### Sepsis Case Database Compilation

To identify dogs with presumptive sepsis, electronic medical records (EMR) of the Cornell University Hospital for Animals from 01/2008 to 12/2018 were queried for dogs with any of 109 diagnosis codes associated with an infection that could lead to sepsis, for example, pneumonia, pyometra or pyelonephritis (Supplementary Data S1). For each patient, case identification number (ID), age, breed, sex, diagnosis, temperature, heart rate, respiratory rate, total leukocyte, segmented neutrophil and band neutrophil counts, discharge date, and outcome (alive, death, euthanasia) were extracted automatically. The resulting database was manually reviewed for patients with incomplete records, undocumented physiologic parameters, multiple visits, multiple or presumptive diagnoses. Additional data were extracted manually from medical records as needed to complete each entry. One author (VC) was responsible for adjudicating the inclusion or exclusion of records from patients with presumptive diagnoses including reading available medical history, physical examination findings, laboratory and pathology data, and discharge statements. Using automated data processing with manual case review as necessary, the presence or absence of presumptive sepsis was then determined for each patient. Sepsis was presumed based on presence of a documented or highly suspected infection accompanied by two or more criteria indicative of the systemic inflammatory response syndrome (SIRS) within the same hospital visit. The SIRS criteria used in this study were: temperature <100.6°F or >102.6°F, heart rate ≥120 beats per minute, respiratory rate ≥40 breaths per minute (23), white blood cell count >16 × 10^3^/μL or <6 × 10^3^/μL, or >3% band neutrophils (24). Respiratory rate was not used as a SIRS criterion for patients for which “panting” was recorded in lieu of a numerical respiratory rate. These patients were still required to satisfy two of the SIRS criteria from the other available parameters. In patients for which multiple sets of physiologic parameters were recorded for a single visit, SIRS was considered to be present if any set of vital parameters collected at a single time point met the above SIRS criteria and the leukocyte or band neutrophil counts from any complete blood count during the patient's hospitalization also met criteria. Patients without a complete blood count were still eligible for inclusion if two SIRS criteria were met by abnormalities of vital parameters alone. Patients with insufficient available EMR data to confirm the presence of sepsis were excluded from the study. Final diagnoses for hypophosphatemia and for presumptive sepsis were allocated based on body systems (Supplementary Data 2,3).

### Hypophosphatemia Case Database Compilation

The electronic database from the institution clinical pathology laboratory containing all canine serum biochemistry analyses from 01/2008 to 12/2018 was queried to obtain all biochemistry profiles from dogs with serum phosphate values below the local reference interval (2.7–5.4 mg/dL) in addition to the associated patient ID. No attempt was made to distinguish hypophosphatemia that was present at the time of admission vs. that which developed in hospital. The hospital EMR system was then queried using these patient ID numbers to obtain patient sex, breed, date of birth, body weight, visit date, main problem, diagnosis, and outcome. If multiple biochemistry profiles were analyzed for the same patient during the same hospitalization period, the lowest phosphate concentration measured was recorded and used for subsequent analyses. Diagnosis and main problems were individually reviewed for possible diagnoses associated with infection that could lead to sepsis (per Supplementary Data S1). For this subset of patients with a diagnosis of infection, temperature, heart rate, respiratory rate, and leukocyte counts were manually recorded from the hospital EMR system. The two final databases (hypophosphatemia and presumptive sepsis) were then compared using the patient ID to identify patients with both hypophosphatemia and presumptive sepsis. As a final check, the medical records for these cases were re-reviewed to ensure that the recorded hypophosphatemia and presumptive sepsis were during the same hospitalization period and to compare the dates of phosphate measurement and satisfaction of SIRS criteria.

### Statistical Analysis

Variables were assessed for normality using the D'Agostino-Pearson test. Continuous patient variables were compared between groups using the Mann-Whitney *U* test with a *post hoc* Bonferroni correction for multiple comparisons (*n* = 6). Within the presumptive sepsis group, phosphate concentrations were compared between survivors and non-survivors by box-whisker plots and using the Mann-Whitney *U* test. Additionally, to quantify the association between outcome and the degree of deviation of patient phosphate from normal, we calculated the delta phosphate value as the absolute numeric difference between the midpoint of the reference interval and the measured patient phosphate value (delta-phosphate), and compared this value between survivors and non-survivors (25). Differences in frequency between categorical variables were analyzed using contingency tables, calculation of Fisher's exact test or Chi^2^, and estimation of odds ratios. Specifically, Fisher's exact test was used to compare the frequency of hypophosphatemia in dogs with and without presumptive sepsis, to compare frequency of non-survival (death or euthanasia) in dogs with hypophosphatemia with and without presumptive sepsis, and to compare frequency of non-survival in dogs with presumptive sepsis with and without hypophosphatemia. In addition, analysis of outcome in dogs with phosphate concentrations <2.0 mg/dL, representing the lowest 20% of measured phosphate concentrations was performed using Fisher's exact test. Receiver operating characteristic curve analysis was used to evaluate the ability of phosphate concentrations to discriminate survivors from non-survivors in dogs with hypophosphatemia and presumptive sepsis. All analyses were performed using commercial software (Prism 9, GraphPad, La Jolla, CA).

## Results

### Incidence of Hypophosphatemia and Presumptive Sepsis

**Figure 1:**
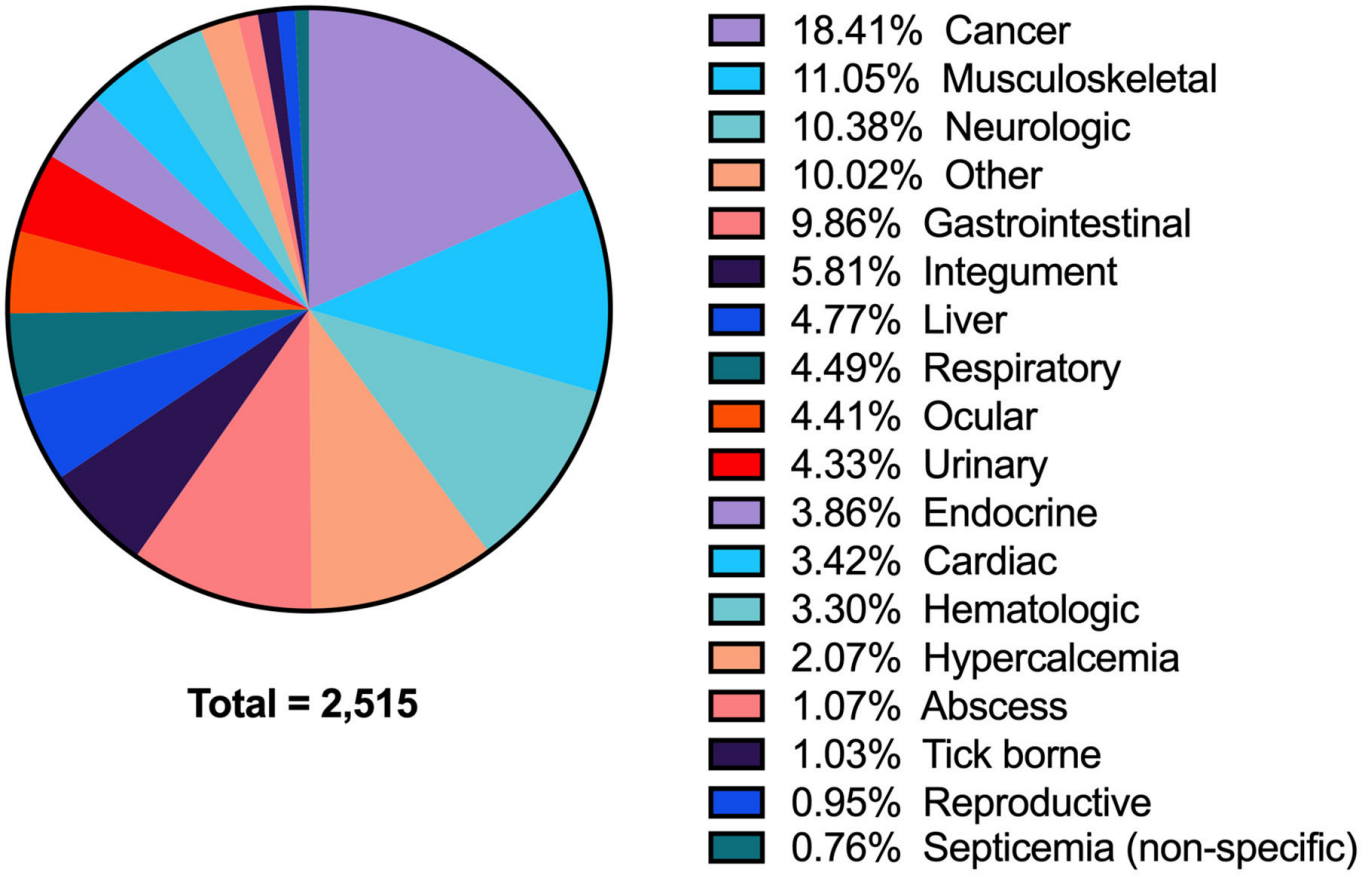
A pie-chart detailing all of the associated diagnoses in dogs with hypophosphatemia. All diagnoses are grouped by anatomic body system (see Supplementary Data S2 for detailed list of individual diagnoses).

Between 2008 and 2018, 47,992 biochemistry profiles from 23,752 dogs were identified. Of those, there were 3,156 records with a phosphate concentration below the reference interval. These 3,156 records were from 2,515 unique dogs, indicating that 641 cases had more than one documented value of hypophosphatemia. After eliminating duplicate analyses, the period prevalence of hypophosphatemia on a per dog basis during the 11-year study period was 10.6% (2,515/23,752). The associated diagnoses for these 2,515 patients are shown in Figure 1; with the most commonly associated conditions being cancer (18.4%), musculoskeletal disorders, including wounds (11.1%), neurologic diseases (10.4%), and gastrointestinal disorders (9.9%). Sepsis was presumptively diagnosed in 10.7% (268/2,515) of dogs with hypophosphatemia.

**Figure 2:**
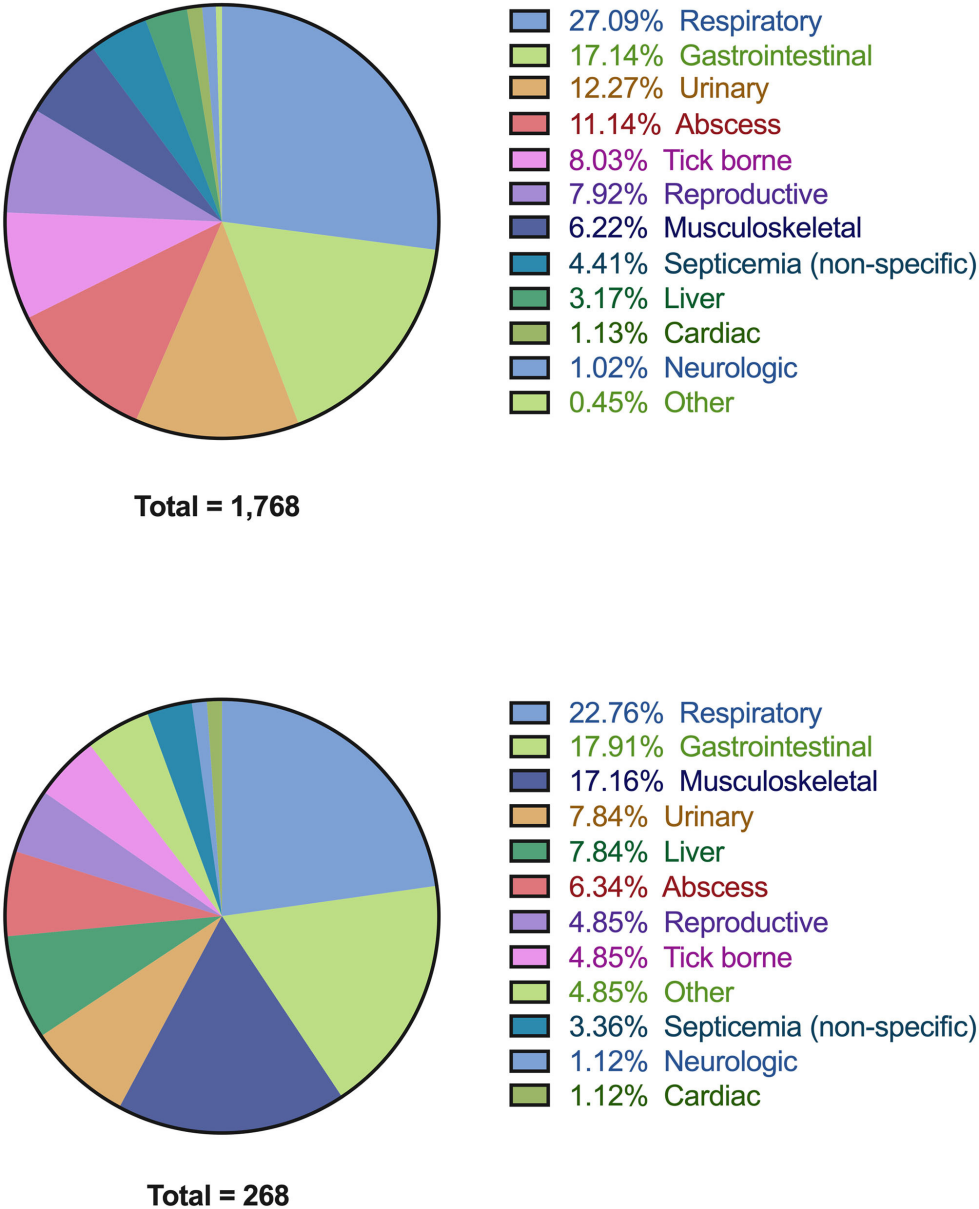
Pie-charts detailing all of the associated diagnoses in dogs with presumptive sepsis. All diagnoses are grouped by anatomic body system (see Supplementary Data S3 for detailed list of individual diagnoses). **(A)** Associated diagnoses in all dogs with presumptive sepsis (*n* = 1,768). **(B)** Associated diagnoses in dogs with presumptive sepsis and hypophosphatemia (*n* = 268).

Within the study period, 4,406 case records were identified with a diagnosis of infection, of which 809 records were incomplete and were excluded from further analysis. Of the remaining 3,597 cases, 1,798 records from 1,768 unique patients satisfied ≥2 SIRS criteria and were presumptively diagnosed with sepsis. Of the 1,768 patients, 1,233 cases had a contemporaneous phosphate concentration available. Of these 21.7% (268/1,233) of cases were hypophosphatemic. The lowest phosphate concentration was measured a median of 0 (IQR 0-1) days from when dogs satisfied SIRS criteria. The underlying locations of the disorder causing presumptive sepsis in all dogs and the dogs with presumptive sepsis and hypophosphatemia are summarized in Figure 2. The most common sites of presumptive sepsis were the respiratory tract (27.1%), gastrointestinal tract (17.1%), urinary tract (12.3%). In dogs with presumptive sepsis and hypophosphatemia, the pattern of sites affected was similar, with the three most common sites being the respiratory tract (22.8%) gastrointestinal tract (17.9%), and the musculoskeletal system, including wounds (17.2%).

### Patient Demographics

A summary of patient demographics for all the dogs in the study, categorized by phosphate concentration and disease is presented in Table 1. The SIRS criteria parameters and outcomes for the presumptive sepsis cases are summarized in Table 2. After correction for multiple comparisons, dogs with presumptive sepsis and hypophosphatemia had lower median total leukocyte counts compared to dogs with presumptive sepsis without hypophosphatemia 14.7 × 10^3^/μL (IQR 8.4–22.5) vs. 18.7 × 10^3^/μL (IQR 12.5–27.7) (*P* < 0.001). This difference in total leukocyte count was likely due to lower median neutrophil counts in dogs with presumptive sepsis and hypophosphatemia compared to dogs with presumptive sepsis without hypophosphatemia: 11.7 × 10^3^/μL (IQR 6.2–17.9) vs. 14.9 × 10^3^/μL (IQR 8.9–22.1) respectively, (*P* < 0.001).

**Table 1:** Patient demographics for all dogs categorized by disease and phosphate concentration.

	**All hypophosphatemia** **(*n* = 2,515)**	**Hypophosphatemia without presumptive sepsis** **(*n* = 2,247)**	**All presumptive sepsis cases** **(*n* = 1,768)**	**Presumptive sepsis with known phosphate** **(*n* = 1,233)**	**Presumptive sepsis with hypophosphatemia** **(*n* = 268)**	**Presumptive sepsis with N or H phosphate** **(*n* = 965)**
Age (years)	8 (5–10)	8 (5–10)	6 (2–10)	7 (3–10)	6 (4–10)	7 (3–10)
Sex (*n*)						
F	108	81	284	181	27	154
FS	1,145	1,029	631	472	116	356
M	127	111	244	152	16	136
MC	1,135	1,026	609	428	109	319
Bodyweight (kg)	17.8 (7.3–31.5)	17.8 (7.3–31.6)	22.4 (8.5–34.6)	22.9 (8.4–35.0)	16.3 (7.2–30.8)	24.0 (9.3–35.9)
Outcome (*n* (%))						
Alive	2,405 (95.6%)	2,178 (96.9%)	1,386 (78.4%)	1,026 (83.2%)	227 (84.7%)	799 (82.8%)
Died	14 (0.6%)	8 (0.4%)	60 (3.4%)	37 (3.0%)	6 (2.2%)	31 (3.2%)
Euthanized	96 (3.8%)	61 (2.7%)	322 (18.2%)	170 (13.8%)	35 (13.1%)	135 (14.0%)

*Summary statistics for continuous variables age and bodyweight are given as median (interquartile range)*.

*F, female intact; FS, female spayed; H, high; M, male intact; MC, male castrated; N, normal*.

**Table 2:** A summary of vital physiologic parameters, leukocyte counts and outcome in dogs with presumptive sepsis, categorized by phosphate concentration.

	**Presumptive sepsis known Phos** **(*n* = 1,233)**	**Presumptive sepsis Phos-L** **(*n* = 268)**	**Presumptive sepsis** **Phos-N or -H** **(*n* = 965)**	***P*-value** **Phos-L vs.** **Phos-N or -H**	***P*-value** **Bonferroni adjusted**
T (°F)	102 (101–103)	103 (100–103)	102 (101–103)	0.205	1.000
P (bpm)	132 (120–156)	132 (120–160)	130 (120–156)	0.242	1.000
R (rpm)	40 (30–56)	36 (30–52)	40 (30–60)	0.044	0.264
WBC (×10^3^/μL)	18.1 (11.5–26.8)	14.7 (8.4–22.5)	18.7 (12.5–27.7)	<0.0001	**<0.0001**
Neutrophils (×10^3^/μL)	14.1 (8.0–21.4)	11.7 (6.2–17.9)	14.9 (8.9–22.1)	<0.0001	**<0.0001**
Bands (×10^3^/μL)	0.4 (0.0–1.6)	0.4 (0.0–1.8)	0.3 (0.0–1.5)	0.207	1.000
Outcome (*n* (%))					
Alive	1,026 (83.2%)	227 (84.7%)	799 (82.8%)	0.642	-
Dead	37 (3.0%)	6 (2.2%)	31 (3.2%)		
Euthanized	170 (13.2%)	35 (13.1%)	135 (14.0%)		

*Summary statistics for continuous variables are given as median (interquartile range)*.

*T, temperature; °F, degrees Fahrenheit; P, pulse rate; bpm, beats per minute; R, respiratory rate; rpm, respirations per minute; WBC, leukocyte count; Neutrophils, mature neutrophil count, Bands, band neutrophil count. P-values highlighted in bold text are significant at P < 0.05 after correction for multiple comparisons*.

**Figure 3:**
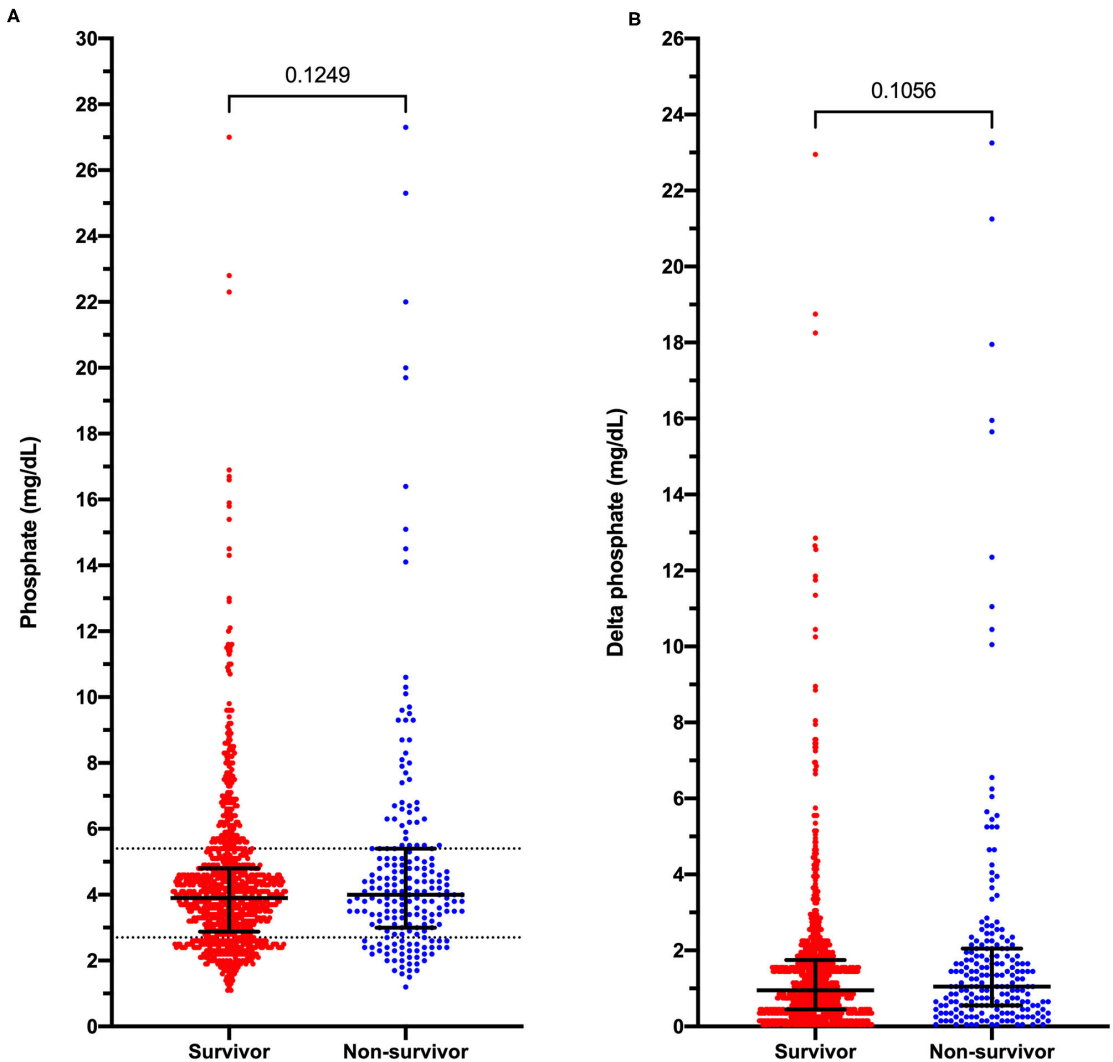
Dot plots of phosphate concentrations in dogs with presumptive sepsis categorized by outcome into survivors and non-survivors (death and euthanasia). **(A)** Measured serum phosphate concentrations in dogs with presumptive sepsis. **(B)** Delta-phosphate concentrations (deviations from the reference interval midpoint) in dogs with presumptive sepsis. Solid black lines represent the median and interquartile range. Dotted horizontal lines on panel A represent the institution reference interval values. *P*-values were calculated using the Mann-Whitney *U* test.

Hypophosphatemia was more prevalent in dogs with presumptive sepsis compared to the overall population of dogs that underwent serum biochemistry testing (21.7% vs. 10.2%; OR 2.44 [95% CI 2.12–2.81]; *P* < 0.0001). Septic hypophosphatemic dogs were more likely to die or be euthanized compared to hypophosphatemic dogs without presumptive sepsis (15.3 vs. 3.1%; OR 5.70 [95% CI 3.76–8.52]; *P* < 0.0001). However, hypophosphatemia was not associated with an increased risk of death or euthanasia in dogs with presumptive sepsis. The mortality rate in dogs with presumptive sepsis and hypophosphatemia was 15.3 vs. 17.2% in dogs with presumed sepsis that were not hypophosphatemic; OR 0.87 [95% CI 0.60–1.26]; *P* = 0.518. Likewise, hypophosphatemic septic patients were not at an increased risk of death (after exclusion of euthanasia) compared to dogs with presumptive sepsis without hypophosphatemia (2.6 vs. 3.7%; OR 0.54 [95% CI 0.30–1.63]; *P* = 0.544). Within the presumptive sepsis group, no difference was found in the median phosphate concentration between survivors and non-survivors (*P* = 0.125), or the median delta-phosphate concentration (*P* = 0.106) (Figure 3). Analyses of outcome in dogs with phosphate concentrations <2.0 mg/dL, that represented the lowest 20% of phosphate values recorded did not identify an association between very low phosphate concentrations and outcome, OR 1.073 (0.514–2.189), *P* = 0.847. Receiver operating characteristic curve analysis indicated that phosphate concentrations were not discriminating for outcome in dogs with presumptive sepsis and hypophosphatemia; AUROC 0.534 (95% CI 0.489–0.579), *P* = 0.125.

## Discussion

This study aimed to determine the incidence of hypophosphatemia in dogs, and in dogs with presumed sepsis in particular, and to determine if hypophosphatemia was associated with outcome in dogs with presumptive sepsis. The overall period prevalence of hypophosphatemia in dogs that underwent serum biochemistry testing at our institution was 10.6%. Although this suggests that hypophosphatemia is common in dogs assessed at our hospital, it is lower than the prevalence of other electrolyte abnormalities we have previously reported. Analyses of data derived from a previously published study investigating the impact of electrolyte disturbances on outcome at our institution (25), revealed prevalence of hypokalemia, hyponatremia, hypochloremia and ionized hypocalcemia to be 34.8, 29.5, 22.2, and 16.8%, respectively. In humans, the overall prevalence of hypophosphatemia in asymptomatic patients is only 5% (26). Critical illness associated with head trauma, DKA, alcoholism, major hepatic or cardiac surgery, and sepsis has been documented to have an increased prevalence of hypophosphatemia in humans (3, 4).

Hypophosphatemia can result from decreased intestinal absorption, redistribution of extracellular phosphorus to the intracellular compartment, increased renal excretion, or combinations of these phenomena (27). In sepsis, hypophosphatemia most commonly occurs via redistribution of inorganic phosphate from the extracellular fluid into cells. This can occur secondary to increased catecholamine concentrations that stimulate glycolysis and the formation of phosphorus-containing metabolic intermediates. Additionally, critically ill patients may suffer from hyperglycemia necessitating the administration of insulin that drives the transport of phosphate into cells (4, 28). Respiratory alkalosis due to hyperventilation, which can be present during sepsis, heatstroke, and hepatic disease, can also decrease intracellular carbon dioxide, stimulate phosphofructokinase activity and glycolysis and cause phosphate to enter cells (29). Chronic phosphate depletion due to decreased phosphate intake resulting from malnutrition/malabsorption and increased loss through increased renal excretion in response to metabolic acidosis and drugs including diuretics and glucocorticoids have also been reported in critically ill patients with hypophosphatemia (1, 2, 26).

Hypophosphatemia in dogs has not been extensively studied. To our knowledge, our study is the first to document the prevalence of hypophosphatemia in a large population of dogs and to evaluate the associated disease processes. Two case series have documented hypophosphatemia associated with the treatment of diabetes mellitus and DKA in six dogs and two cats (8, 9). Hypophosphatemia has also been reported in dogs with refeeding syndrome and in hyperparathyroidism (11, 30). In the present study, hypophosphatemia was most frequently seen in dogs with cancer, musculoskeletal disorders, and disease of the neurologic and gastrointestinal systems. In humans, hypophosphatemia is well-documented in patients with malignant lymphoma, leukemia, and other tumors (31–33). While hypophosphatemia has been documented in dogs with diabetes, endocrine disorders were uncommonly associated with hypophosphatemia in the present study (3.9%). Hyperparathyroidism is a frequent cause of hypophosphatemia in dogs, with almost all dogs with primary hyperparathyroidism having a serum phosphorus below or in the lower half of the reference range in one study (11). Although hypophosphatemia is very prevalent among dogs with hyperparathyroidism, in our study only a small number of dogs were diagnosed with hyperparathyroidism (categorized under hypercalcemia). Future studies might focus on confirming the incidence, prognostic significance, and potential treatment of hypophosphatemia in dogs with cancer and gastrointestinal disorders.

Informed by the human medical literature, we hypothesized that hypophosphatemia is more prevalent in dogs with presumptive sepsis than in dogs with other disease processes. Indeed, this was the case, with our study showing 21.7% of dogs with presumptive sepsis also had hypophosphatemia documented during their hospital stay. Comparably, the prevalence of hypophosphatemia in human septic patients is higher than in other critically ill patients. A retrospective observational study in a human intensive care unit documented 23% of their critically ill patients to hypophosphatemic (13), while hypophosphatemia has been described in up to 60–80% of human septic patients (12, 34). The lower prevalence of hypophosphatemia in our septic population may be multifactorial. A difference in overall illness severity between the dogs with presumptive sepsis enrolled here and those reported in human studies might be a factor. The present study used a definition of sepsis based on the 2001 Society of Critical Care Medicine consensus conference (35) and used the presence of SIRS due to infection to define the syndrome. In humans, it is recognized that the SIRS criteria have limited specificity and sensitivity for sepsis (36, 37) and should only be applied to patients with compatible history, clinical signs, and physical examination findings. In the present study, we attempted to decrease the likelihood of misdiagnosis or misapplication of the SIRS criteria by pre-specifying a list of potential infectious diseases that might cause sepsis and combined that with careful manual clinical record review. However, it remains possible that our use of the SIRS criteria in a retrospective study might be overly sensitive.

In the present study, patients could satisfy the SIRS criteria with complete blood count abnormalities identified at times distinct from identification of disturbances in vital parameters. This was necessary due to the large scale and retrospective nature of the present study. This more liberal approach to application of the SIRS criteria might have diminished their specificity for sepsis diagnosis and hence might have led to inclusion of patients that with lesser illness severity than other canine sepsis populations reported. The present study used a higher respiratory rate cutoff than in other comparable studies. We set a rate of 40 breaths per minute as our respiratory criterion to reduce the likelihood that factors such as pain or anxiety might inadvertently lead to inclusion. Some investigators have used a respiratory rate of 30 to define SIRS (38), but it has been demonstrated that resting respiratory rates of normal dogs can be up to 30 breaths per minute (39). A rate of 40 breaths per minute was therefore used in the present study. This might have diminished sensitivity at the expense of specificity.

In human medicine, sepsis was recently redefined to enhance the specificity of the clinical criteria used to identify the syndrome (40). The Sepsis-3 definitions incorporate a rapid organ failure assessment score, that has yet to be robustly examined in veterinary medicine and used big data analyses to tie definitions to mortality estimates. As such, the Sepsis-3 definitions cannot be directly applied to dogs, and hence using the SIRS criteria remains the best available strategy. The 2016 Sepsis-3 definitions eliminated the prior term severe sepsis and hence the new human sepsis definitions likely identify patients with greater illness severity than was present in the dogs included in our study. An equivalent redefinition of sepsis in veterinary medicine had not been undertaken, but might in turn identify a sicker population than that included here.

Dogs in the present study had various underlying infections, most frequently the respiratory, gastrointestinal, and urinary tracts, while the two most common infections of septic patients with hypophosphatemia were the respiratory tract and gastrointestinal tract, with musculoskeletal conditions and wounds the third most common source. This suggests a concordance of conditions that cause sepsis and hypophosphatemia. Overall, the distribution of underlying infections in our population of dogs with presumptive sepsis was similar to various previous studies (14, 21, 22, 41–43).

In humans, hypophosphatemia is particularly prevalent in sepsis due to Gram-negative bacterial infections (2). We did not subdivide our dogs with presumptive sepsis into those suffering from Gram-negative bacterial infections and hence a higher prevalence of hypophosphatemia might have been present in those dogs. The design of the present study prevented us from assessing the effect of therapy or supplementation on phosphate concentrations. For instance, use of insulin has been established as a risk factor for hypophosphatemia in dogs and cats (8, 9). In critically ill humans with sepsis, tight glucose regulation using insulin to manage disease-associated hyperglycemia has been commonly practiced (44). It is unknown how many of the dogs in the present study received insulin, but insulin is rarely used to manage hyperglycemia associated with critical illness in our institution and hence the percentage is likely small. This might have reduced the frequency of hypophosphatemia in our study relative to the human literature.

Hypophosphatemia in humans with sepsis is a marker of illness severity and hence might be expected to be more prevalent in a population with a high degree of illness severity. Scoring systems for estimating illness severity exist in veterinary medicine, but they are challenging to apply to large populations generated by retrospective record analysis because they frequently require measurement of parameters that are not universally collected or measured such as mean arterial blood pressure, lactate concentration, body cavity fluid scores or oxygenation indices (45–47). A prospective study might enable a more uniform collection of these data, but it would be challenging to prospectively recruit and enroll the number of dogs that were included in the present study in a timely manner. In the present study, many dogs within the presumptive sepsis group did not have a measured phosphate concentration and hence were excluded from the final calculations of frequency and from comparisons of outcome. There are likely to have been various reasons why these patients did not have a phosphate concentration measured, but several factors might have tended to exclude patients with more severe disease including patients that died or were euthanized shortly after diagnosis and those patients where financial constraints might have limited both access to preventative veterinary health care and precluded serum biochemistry analysis.

The present study found that among dogs with hypophosphatemia, dogs with presumptive sepsis were less likely to survive than dogs with hypophosphatemia associated with other disease processes. This is to be expected—it is well-recognized that sepsis is associated with high mortality in dogs (14, 18, 21, 22, 47, 48). Hypophosphatemia in humans with sepsis is a risk factor for mortality, particularly when severe (49). On this basis, we hypothesized that hypophosphatemia in dogs with presumptive sepsis is associated with increased mortality. Surprisingly, and counter to our hypothesis, dogs with presumptive sepsis and hypophosphatemia did not have higher mortality rates than dogs with presumptive sepsis and normal or high phosphate concentrations. This may be because we chose to include patients with any value of phosphate lower than the reference interval (<2.7 mg/dL). Hypophosphatemia might be a risk factor for mortality predominantly when serum phosphate level is severely low as serum phosphate levels do not reflect total body phosphate. Mild hypophosphatemia may have a limited influence on physiology (3), and hence perhaps on outcome. By contrast, severe hypophosphatemia has been shown to cause numerous detrimental effects such as increased cardiac arrhythmias, decreased myocardial function, hemolysis, decreased oxygen delivery to tissues, and respiratory failure (3, 6, 50). These complications may explain an increased risk of mortality with severe hypophosphatemia.

Our study population did not contain any dogs with presumptive sepsis and severe hypophosphatemia per the human medical definition (phosphate <1.0 mg/dL). The lowest recorded phosphate concentration in any dog with presumptive sepsis in the present study was 1.1 mg/dL. Analyses of outcome in dogs with phosphate concentrations <2.0 mg/dL, that represented the lowest 20% of phosphate values recorded did not identify an association between very low phosphate concentrations and outcome. In addition, receiver operating characteristic curve analysis also did not suggest that phosphate concentrations were discriminating for outcome in dogs with presumptive sepsis and hypophosphatemia. This is consistent with the comparable delta-phosphate values in non-survivors vs. survivors.

Owing to the retrospective nature of the study, we could only analyze patients for which a phosphate concentration was measured and only at that single time point. Hence missing data or failure to identify the nadir might have affected the strength of association identified. A prospective study measuring phosphate concentrations serially might be better able to identify an association between outcome and the nadir phosphate concentration or the change in phosphate over time.

Our study has some limitations. Throughout the manuscript, we refer to presumptive sepsis, rather than sepsis to reflect the degree of uncertainty that is inherent to our methodology. Due to the retrospective nature of the study, it was not possible to be certain that SIRS criteria were satisfied at the exact time the patients had the illness that was recorded as their final diagnosis, although this is considered highly likely. Similarly, owing to the retrospective design, only those data recorded in the EMR could be analyzed. A large number of records were excluded due to missing data including physiologic parameters, complete blood counts, or serum biochemistry values. These data may not have been missing at random. A lack of true randomness in the patients from which values were missing might introduce bias. For instance, if data points or records were more likely to be missing from very sick patients or those with financial limitations, this might affect our assessments of prevalence and our comparisons between groups. Our study is considerably larger than most analyses of canine sepsis and of hypophosphatemia in veterinary medicine, and hence the effect of missing data might be lessened, but cannot be eliminated. Due to the size of the patient populations, it was not feasible to individually review all medical records of dogs with hypophosphatemia to determine if they were suffering from a critical illness other than sepsis, or whether the primary or secondary disease processes had been previously associated with hypophosphatemia. When reporting causes of hypophosphatemia, we did not evaluate the presence of drugs that were administered that can lower phosphate levels or evaluate blood gas analyses for signs of respiratory alkalosis. We also were not able to standardize the date of hypophosphatemia, electing to use the lowest phosphate concentration recorded during a patient's visit. The median (IQR) time interval between this measurement and the date dogs satisfied SIRS criteria was 0 (0–1) days, suggesting that this likely had minimal impact on the association between hypophosphatemia and outcome. Lastly, like many veterinary studies, bias linked to euthanasia and long term follow up is missing. Analyses of outcomes at 30- or 90-days might have altered the conclusions drawn from group comparisons.

In summary, the overall prevalence of hypophosphatemia in dogs in this study was 10.6% which although common was less frequent than that reported for other electrolytes in a comparable population. Presumed sepsis was commonly associated with hypophosphatemia in dogs. In dogs with hypophosphatemia, presumptive sepsis was a risk factor for non-survival, but hypophosphatemia was not a risk factor for non-survival in dogs with presumptive sepsis. Future studies might evaluate the impact of illness severity and therapy on phosphate concentrations in dogs with sepsis.

## Data Availability Statement

The raw data supporting the conclusions of this article will be made available by the authors, without undue reservation.

## Ethics Statement

This study was exempted from local institutional Ethics Committee approval. Ethical review and approval was not required for this study because it presents a retrospective analysis of patient data collected as part of clinician-driven care provided to patients at the institution hospital. For the same reason, additional written informed consent for participation was not obtained from owners. No client or patient identifying information is presented.

## Author Contributions

VC collected and analyzed data and wrote the manuscript. RG conceived the study, analyzed data, and edited the manuscript. SR and AB collected and analyzed data. JM analyzed data and edited the manuscript. All authors contributed to the article and approved the submitted version.

## Conflict of Interest

The authors declare that the research was conducted in the absence of any commercial or financial relationships that could be construed as a potential conflict of interest.
