# Supplementary material for: Hypophosphatemia in Dogs With Presumptive Sepsis: A Retrospective Study (2008–2018)
Source: Front Vet Sci. 2021 Mar 8;8:636732. doi: 10.3389/fvets.2021.636732 (PMC7982394; doi:10.3389/fvets.2021.636732)
Supplement: Supplementary file 1 [file Data_Sheet_1.PDF]

Infection Queried Diagnosis List

Abscess (and all the subcategories)
  a. Cerebral
  b. Lung
  c. Foot
  d. Corynebacterium
  e. Liver
  f. Lymph Node
  g. Peritoneal
  h. Mesenteric
  i. Prostatic
  j. Orbital
  k. Retroperitoneal
  l. Retropharyngeal
  m. Skin-fungal
  n. Skin-bacterial
  o. SQ-bacterial
  p. Tooth
  q. SQ-fungal
  r. Anal

Anaerobic infection
Anal sac infection
Anaplasmosis
Arthritis – septic
Babesiosis
  a. Babesiosis – canine

Bacterial pneumonia
Blastomycosis
Canine distemper
Canine distemper virus
Canine erhlichiosis
Canine granulomatous hepatitis
Canine hepatitis
Canine neosporosis
Canine Rocky mountain spotted fever
Cellulitis – aerobic
Cellulitis – anaerobic
Choangiohepatitis
Cholecystitis
Cholecystitis – emphysematous
Cholecystitis- necrotizing
Clostridial - infection
Discospondylitis – bacterial
Discospondylitis – fungal
Empyema
Endocarditis
  a. Endocarditis – Aortic
  b. Endocarditis – Mitral
  c. Endocarditis – Pulmonic
  d. Endocarditis – Tricuspid

Enteritis – parvovirus
Fungal pneumonia
Hepatitis
  a. Canine granulomatous hepatitis
  b. Canine hepatitis
  c. Hepatitis - acute

Leptospirosis
Lyme borreliosis
Lyme disease
Meningoencephalomyelitis – bacterial
Metritis – septic
MRSA infection
Mycobacterial infection
Mycoplasma infection
Myelitis
  a. Myelitis – bacterial
  b. Myelitis – distemper
  c. Myelitis – protozoal – neospora canis
  d. Myelitis – protozoal – toxoplasma canis
  e. Myelitis – rickettial – RMSF

Neonatal septicemia
Oral Mass – infectious
Osteomyelitis – bacterial
Osteomyeltis – fungal
Osteomyelitis – infectious
Papillomavirus infection - canine
Parvovirus
Parvovirus encephalitis
Pericardial effusion – infectious
Peritonitis – infectious
Peritonitis – septic
Pleuropneumonia
Pneumonia
  a. Bacterial pneumonia
  b. Pneumonia
  c. Pneumonia – aspiration
  d. Pneumonia – bacterial
  e. Pneumonia – bordetella bronchiseptica
  f. Pneumonia - fungal – blastomycosis
  g. Pneumonia – interstitial
  h. Pneumonia – lung worms
  i. Pneumonia - mycoplasma
  j. Pneumonia – protozoal – pneumocystis carinii
  k. Viral pneumonia

Pyelonephritis
Pyometra
  a. Pyometra - stump

Pyothorax
Prostatitis
Septic arthritis
Septicemia
Sinusitis – bacterial
Sinusitis – fungal
Upper respiratory infection
Urinary tract infection
Vaginitis
Vestibular disease- bacterial
Wound infection
