# Supplementary material for: Hypophosphatemia in Dogs With Presumptive Sepsis: A Retrospective Study (2008–2018)
Source: Front Vet Sci. 2021 Mar 8;8:636732. doi: 10.3389/fvets.2021.636732 (PMC7982394; doi:10.3389/fvets.2021.636732)
Supplement: Supplementary file 2 [file Data_Sheet_2.PDF]

Abscess
Anal Gland
Cerebral
Corynebacterium
Foot
Liver
Lung
Lymph Node
Mesenteric
Orbital
Peritoneal
Prostatic
Retroperitoneal
Retropharyngeal
Skin – bacterial
Skin – fungal
SQ – bacterial
SQ – fungal
Tooth

Cancer
Abdominal mass
Adenocarcinoma
Adenoma
Adrenal mass
Ameloblastoma – oral acanthomatous
Carcinoma
Fibrosarcoma
Hemangiosarcoma
Leukemia
Lipoma
Lymphoma
Mass – undiagnosed – primary
Mast cell tumor
Melanoma
Neoplasia
Osteosarcoma

Cardiac
Arrhythmia
Cardiomyopathy – dilated – idiopathic
Chronic myxomatous degeneration
Endocarditis
Heart failure – congestive
Myocarditis

Endocrine
Atypical Addison's
Diabetes mellitus
Diabetic ketoacidosis
Exocrine pancreatic insufficiency
Hyperadrenocorticism
Hyperthyroidism
Hypoadrenocorticism
Hypothyroidism

Gastrointestinal
Acute hemorrhagic diarrhea syndrome
Diarrhea
Esophagitis
Foreign body
Gastric dilatation – volvulus
Inflammatory bowel disease
Intestinal foreign body
Megaesophagus
Pancreatitis
Parvovirus
Peritonitis – septic
Protein losing enteropathy
Septicemia; AHDS
Vomiting

Hematologic
Anemia
Coagulopathy
Epistaxis
Immune mediated hemolytic anemia
Immune mediated thrombocytopenia
Neutropenia

Hypercalcemia
Hypercalcemia
Hyperparathyroidism

Integument
Adverse drug reaction – cutaneous
Alopecia
Atopy
Canine demodicosis – adult-onset
Dental calculus
Dermatitis
Folliculitis – staphylococcal

Liver
Acute liver failure
Biliary obstruction
Canine hepatitis
Cholangiohepatitis
Cholangitis
Cholecystitis
Copper associated – hepatopathy
Gallbladder mucocoele
Hepatic microvascular dysplasia
Portosystemic shunt – multiple acquired

Musculoskeletal
Bite wound(s)
Abrasion
Arthritis
Arthritis – septic
Cellulitis

Cranial cruciate injury – stifle
Degloving injury
Fracture
Hernia
Hip dysplasia
Laceration
MRSA infection
Osteomyelitis
Wound dehiscence
Wound infection

Neurologic
Meningoencephalomyelitis – bacterial
Abiotrophy – cerebellar
Atrophy – cerebellar cortical
Discospondylitis
Intervertebral disc disease
Myelitis
Myelopathy
Seizures
Vestibular disease

Ocular
Aphakia
Atrophy – iris – generalized
Blindness
Cataract
Glaucoma
Pseudophakia

Other
Anorexia
Anxiety
Apparently healthy
Canine distemper
Canine neosporosis
Clostridial infection
Heat stroke
Incomplete record
Lab only
Myiasis
Mycobacterial infection
Mycoplasma infection
Open
Sinusitis

Reproductive
Abortion – *Neospora*
Blepharitis
Mastitis
Metritis
Prostatitis
Pyometra
Vaginitis

Respiratory
Blastomycosis
Brachycephalic airway syndrome
Bronchitis
Coughing
Pneumonia
Pyothorax
Upper respiratory infection

Septicemia (nonspecific)
Neonatal septicemia
Septicemia – nonspecific

Tick borne
Anaplasmosis
Babesiosis – canine
Canine ehrlichiosis
Canine rocky mountain spotted fever
Lyme disease

Urinary
Calculi – cystic
Chronic kidney disease
Leptospirosis
Proteinuria
Pyelonephritis
Urinary tract infection
