# Supplementary material for: Hypophosphatemia in Dogs With Presumptive Sepsis: A Retrospective Study (2008–2018)
Source: Front Vet Sci. 2021 Mar 8;8:636732. doi: 10.3389/fvets.2021.636732 (PMC7982394; doi:10.3389/fvets.2021.636732)
Supplement: Supplementary file 3 [file Data_Sheet_3.PDF]

Abscess
Anal gland
Anal sac infection
Cerebral
Corynebacterium
Foot
Liver
Lung
Lymph node
Mesenteric
Orbital
Peritoneal
Prostatic
Retroperitoneal
Retropharyngeal
Skin-bacterial
Skin-fungal
Sq-bacterial
Sq-fungal
Tooth

Cardiac
Endocarditis
Myocarditis

Gastrointestinal
Parvovirus
Peritonitis – septic
Septicemia; AHDS

Liver
Bile peritonitis
Canine hepatitis
Cholangiohepatitis
Cholangitis
Cholecystitis

Musculoskeletal
Arthritis – septic
Bite wound(s)
Cellulitis
Degloving injury
Laceration
Mrsa infection
Osteomyelitis
Wound dehiscence
Wound infection

Neurologic
Discospondylitis
Meningoencephalomyelitis – bacterial
Myelitis
Vestibular disease – bacterial

Other
Canine distemper
Clostridial infection
Mycobacterial infection
Mycoplasma infection
Myiasis
Sinusitis

Reproductive
Abortion – *Neospora*
Mastitis
Metritis
Neosporosis
Prostatitis
Pyometra
Vaginitis

Respiratory
Aspergillosis
Bacterial pneumonia
Blastomycosis
Bronchitis
Pneumonia
Pyothorax
Upper respiratory infection

Septicemia (nonspecific)
Neonatal septicemia
Neutropenia
Septicemia – nonspecific

Tick borne
Anaplasmosis
Babesiosis – canine
Canine ehrlichiosis
Canine rocky mountain spotted fever
Lyme disease

Urinary
Leptospirosis
Pyelonephritis
Urinary tract infection
